# Comparing the longer regimen and the shorter regimen for multidrug-resistant pulmonary tuberculosis patients treated under the programmatic management of drug-resistant tuberculosis

**DOI:** 10.3389/fmed.2025.1645820

**Published:** 2025-08-28

**Authors:** Ashwin Karnan, Ulhas Jadhav, Babaji Ghewade, Vivek Alone

**Affiliations:** Department of Respiratory Medicine, Datta Meghe Institute of Medical Education and Research, Wardha, Maharashtra, India

**Keywords:** multidrug-resistant pulmonary tuberculosis, culture conversion, adverse drug reactions, malnutrition, alcohol, smoking, diabetes mellitus, Human Immunodeficiency Virus (HIV)

## Abstract

Multidrug-resistant tuberculosis is a major public health concern with prolonged infectivity, a complex treatment regimen, and lower treatment success rates. Despite the significant progress made by India in the control of Tuberculosis, it remains the second leading cause of mortality among infectious diseases. Shorter treatment courses for multidrug-resistant tuberculosis (MDR-TB) can enhance patient adherence by decreasing the length of time for medication intake and alleviating the challenges associated with prolonged treatment. Evaluating the effectiveness of various treatment regimens is crucial for identifying the best balance among treatment duration, efficacy, adverse drug effects, and patient adherence. A prospective, observational study on 50 MDR-TB patients was carried out at a tertiary care hospital. The final cure rates were 88% in the shorter regimen and 84% in the longer regimen, with 12% treatment failure in both groups. Both shorter and longer regimens demonstrated comparable efficacy with slightly better adherence in the shorter regimen.

**Clinical trial registration:**

The study was registered in the Clinical Trials Registry-India (Indian Council of Medical Research-National Institute of Medical Statistics), https://ctri.nic.in/, CTRI registration number CTRI/2024/01/061453, registration date 15/1/2024, date of first enrollment is 24/1/2024.

## 1 Introduction

Tuberculosis (TB) is a highly contagious disease that poses a significant global health threat and ranks among the top causes of mortality on a global scale. Before the outbreak of the COVID-19 pandemic, TB held the title as the primary cause of death attributed to a single infectious agent, surpassing even Human Immunodeficiency Virus/Acquired Immunodeficiency Syndrome (HIV/AIDS) in terms of its impact on mortality rates worldwide (1).

Tuberculosis is an airborne infection caused by the Mycobacterium tuberculosis bacillus. It spreads when individuals infected with TB expel bacteria into the air, primarily through coughing. Inhaling even a small quantity of these bacteria can result in infection. While the disease commonly impacts the lungs (pulmonary TB), it can also affect other areas of the body (extrapulmonary TB), such as the pleura, lymph nodes, abdomen, bones, and meninges. Approximately a quarter of the global population is infected with M. tuberculosis, making it a widespread health concern. Despite the availability of potent anti-TB drugs, tuberculosis ranks second only to COVID-19 in causing the highest number of deaths globally from a single infectious agent. India bears a significant portion of the global TB burden. The WHO’s Global Tuberculosis Report 2024 emphasizes the uneven progress in combating TB worldwide, revealing ongoing issues like substantial underfunding. Despite a decline in TB-related fatalities from 1.32 million in 2022 to 1.25 million in 2023, the estimated number of new TB cases increased slightly to 10.8 million in 2023 (2).

Despite the availability of effective anti-TB medication, tuberculosis remains a significant contributor to the high mortality rates attributed to curable infectious diseases. The Revised National Tuberculosis Control Program (RNTCP) has recently formulated the National Strategic Plan (NSP) to eliminate TB by 2025. However, the goal faces a major obstacle in the form of drug-resistant TB (DRTB), which continues to pose a significant public health threat.

Since 1994, the World Health Organization (WHO) has systematically gathered and analyzed data related to resistance to anti-TB medications, with a primary focus on rifampicin-resistant TB (RR-TB) and multidrug-resistant TB (MDR-TB), which involves resistance to both rifampicin and isoniazid. Together, these are termed MDR/RR-TB. New methodologies introduced in 2022 have enabled the estimation of annual new cases of MDR/RR-TB from 2015 onward. In 2023, it was estimated that there were around 400,000 new cases of MDR/RR-TB globally, remaining stable since 2020, with this strain causing approximately 150,000 fatalities. From 2015 to 2023, the percentage of new TB cases classified as MDR/RR-TB declined, from 4.1% to 3.2%, while the proportion among previously treated cases dropped from 20% to 16%. Trends varied by region: while the Americas saw an increase in cases and the South-East Asia region stabilized, there was a consistent decline reported in Africa, the Eastern Mediterranean, and the Western Pacific (3).

In 2023, a handful of countries contributed to more than half of all global MDR/RR-TB cases, with India accounting for 27% and significant contributions from the Russian Federation, Indonesia, China, and the Philippines. Additionally, there were approximately 1.4 million instances of isoniazid-resistant TB, with 19% of MDR/RR-TB cases also exhibiting pre-XDR-TB characteristics (resistance to certain fluoroquinolones). By the end of 2023, 107 countries reported ongoing surveillance data concerning rifampicin resistance, contributing to a comprehensive understanding of drug resistance in TB worldwide (3).

The Union Health Ministry of India has announced a significant advancement in the treatment of Multi-Drug-Resistant Tuberculosis (MDR-TB) with the approval of the BPaLM regimen. This new approach combines four medications: Bedaquiline, Pretomanid, Linezolid, and optionally Moxifloxacin. Research has shown that this regimen is not only safer but also more effective compared to previous treatment options, which typically lasted around 20 months. In contrast, the BPaLM regimen can effectively treat drug-resistant TB in just six months, offering hope to approximately 75,000 patients across the country (4).

The global treatment success rate for MDR/RR TB patients remains low at 57% (5). The standard DR TB regimen for MDR TB under the Programmatic Management of Drug-Resistant TB (PMDT) involves two main options: the Shorter Oral Bedaquiline-Containing Regimen and the Longer Oral Bedaquiline-Containing Regimen. Patients are assessed based on specific criteria, with those eligible for the shorter regimen starting on it, while the rest begin the longer regimen.

The Shorter Regimen lasts 9–12 months, with a 6 months intensive phase of Levofloxacin (Lfx)/Moxifloxacin (Mfx), Clofazimine (Cfz), Pyrazinamide (Z), Ethambutol (E), High dose Isoniazid (Hh), Ethionamide (Eto), followed by a 5 months continuation phase (Lfx/Mfx, Cfz, Z, E). Eligibility for the oral shorter MDR/RR TB regimen includes having rifampicin resistance, MDR/RR resistance with either an INH A mutation or KatG mutation only (not both), absence of FQ resistance, no previous exposure to second-line drugs for over a month, no extensive TB disease, and no severe extrapulmonary TB.

The Longer MDR TB Regimen spans 18–24 months and is categorized into Groups A, B, and C. While the WHO recommends starting with all Group A agents and at least one Group B agent, in India, experts propose initiating treatment with all five drugs from Groups A and B. Group A includes key foundational agents such as Levofloxacin (Lfx), Moxifloxacin (Mfx), Bedaquiline (Bdq), and Linezolid (Lzd), which are vital for the initial phase of treatment. Group B comprises auxiliary medications such as Clofazimine (Cfz) and either Cycloserine (Cs) or Terizidone (Trd), which should be incorporated to improve treatment efficacy. Finally, Group C consists of drugs that can be utilized to enhance the regimen when the medications from Groups A and B are not appropriate (6, 7).

### 1.1 Aims

To study and compare the efficacy of a longer regimen with a shorter regimen in multidrug-resistant pulmonary tuberculosis patients.

### 1.2 Objectives

- To study the sputum smear examination in multidrug-resistant pulmonary tuberculosis patients treated with a longer regimen and a shorter regimen.
- To study the culture negativity with the duration of treatment in patients on longer regimen and shorter regimen in multidrug-resistant pulmonary tuberculosis.
- To study the clinical profile and outcome in patients on these regimens, respectively.
- To study the treatment adherence, adverse reactions, and any other observations that can be seen during this study.

### 1.3 Study design

Prospective, observational, and comparative study.

### 1.4 Study setting

The study was conducted at Acharya Vinoba Bhave Rural Hospital (AVBRH), a tertiary care hospital, attached to Jawaharlal Nehru Medical College, Sawangi, Meghe, Wardha.

### 1.5 Study population

The patients diagnosed with drug-resistant pulmonary tuberculosis receiving care at the outpatient department (OPD) or admitted to the inpatient department (IPD) of the Respiratory Medicine Department and National Tuberculosis Elimination Program (NTEP) DOTS Center at AVBRH, Sawangi (Meghe), who meet the inclusion criteria, will be included in the study.

### 1.6 Inclusion criteria

- All patients diagnosed with multidrug-resistant pulmonary tuberculosis who are under treatment at Acharya Vinoba Bhave Rural Hospital and NTEP DOTS center, AVBRH, Sawangi, (Meghe), Wardha.
- Patient is more than 18 years of age.

### 1.7 Exclusion criteria

- Patients not giving consent for the study.

### 1.8 Study period

December 2022 to November 2024

### 1.9 Sample size


$$\begin{array}{l} \text{Formula}\\ \text{N}={\text{Z}}^{2}\text{p}(1-\text{p})/{\text{d}}^{2} \end{array}$$

Z = z value (e.g., 1.96 for 95% confidence level)

- Z is the level of significance at 5%, i.e., a 5% confidence interval = 1.96
- P is the prevalence of MDR-TB: 3%
- Desired precision = 5%

$$\begin{array}{l} \text{Sample size}=[1.96^{2}]\times 0.03\times (1-0.03)\\ \;=22.81\;(\text{rounding off to }25)\\ \text{ N}=25 \end{array}$$

## 2 Materials and methods

**FIGURE 1:**
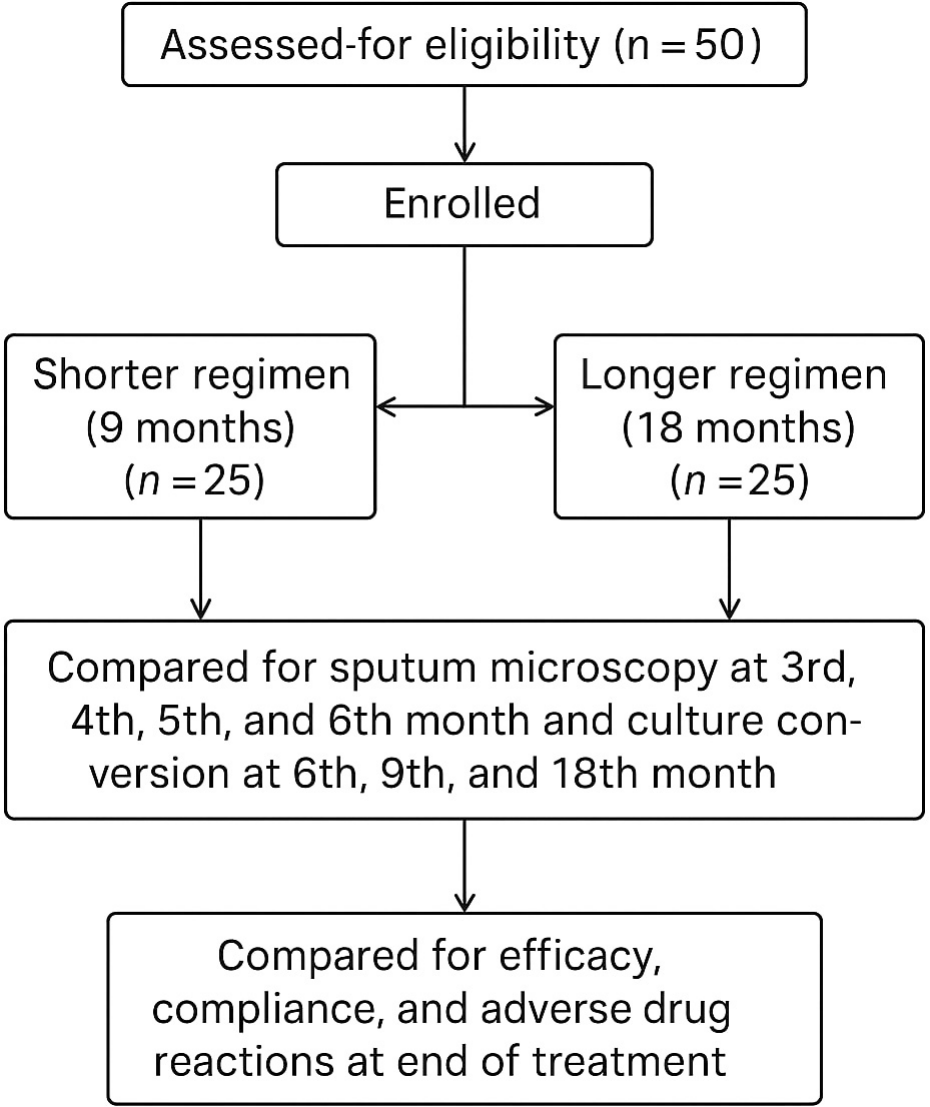
CONSORT flowchart of the study.

1. The study is registered in the Clinical Trials Registry-India with Registration number CTRI/2024/01/061453 and Reference number REF/2023/07/070740.
2. New pulmonary MDR-TB (resistance to both Isoniazid and Rifampicin) patients and those already under treatment for pulmonary MDR-TB at our institute, who satisfied the inclusion and exclusion criteria, were included in this study after obtaining informed consent (Figure 1).
3. The patients were admitted for pre-treatment evaluation and treated on an inpatient basis for the first 7 days, and later were closely observed with regular follow-ups.
4. The patient’s demographic information, detailed history, clinical examination, as well as past illnesses, personal history, and other pertinent history, were obtained.
5. A pre-treatment evaluation was conducted for all patients to identify comorbid conditions, those at risk of adverse effects, those requiring regimen modification, and those with poor outcomes.
6. The patients were divided into two groups. According to the inclusion criteria, one group of 25 patients received a shorter regimen, and the other group of 25 patients received a longer regimen.
7. Shorter all-oral regimen comprised of 9 months of treatment (4 months of intensive phase and 5 months of continuation phase) of:
  Tab. Bedaquiline 400 mg od × 2 weeks followed by 200 mg 3 times/week × 24 weeks
  Tab. Levofloxacin 15–20 mg/kg/day
  Tab. High-dose isoniazid 20–25 mg/kg/day
  Tab. Pyrazinamide 25–30 mg/kg/day
  Tab. Ethambutol 15–20 mg/kg/day
  Tab. Clofazimine 50–200 mg/day
  Tab. Ethionamide 10–15 mg/kg/day
8. Longer oral regimen comprised of 18 months of treatment of:
  Tab. Bedaquiline 400 mg od × 2 weeks followed by 200 mg 3 times/week × 24 weeks
  Tab. Levofloxacin 15–20 mg/kg/day
  Tab. Linezolid 600 mg/day
  Tab. Cycloserine 250–1,000 mg/day
  Tab. Clofazimine 50–200 mg/day
9. Sputum smear examination by fluorescent microscopy was done at the 3^rd^,4^th^, 5^th^, and 6^th^ months. Sputum culture was done at the 3^rd^, 6^th^, 9^th^, 12^th^ months, and at the end of treatment. Sputum smear examination was conducted at our DOTS center, AVBRH hospital. A culture test was done at the Intermediate Research Lab, Nagpur, with the help of the District TB officer and other concerned staff.
10. Both groups were assessed and compared for smear conversion, culture conversion, adverse effects, and treatment outcome (Table 1).

**TABLE 1:** Statistical analysis of the study.

Parameters	Treatment		*P*-value
	Shorter regimen (*n* = 25)	Longer regimen (*n* = 25)	
**Age (years)**	34.36 ± 9.95	48.52 ± 15.57	< 0.001 (1)
**Age group**			0.006 (2)
20–40 years	19 (76.0%)	9 (36.0%)	
41–60 years	6 (24.0%)	11 (44.0%)	
> 60 years	0 (0.0%)	5 (20.0%)	
**Gender**			0.758 (3)
Male	17 (68.0%)	18 (72.0%)	
Female	8 (32.0%)	7 (28.0%)	
**Taluk**			0.784 (2)
Wardha	5 (20.0%)	6 (24.0%)	
Samudrapur	5 (20.0%)	4 (16.0%)	
Arvi	2 (8.0%)	4 (16.0%)	
Hinganghat	3 (12.0%)	3 (12.0%)	
Karanja	1 (4.0%)	3 (12.0%)	
Ashti	2 (8.0%)	1 (4.0%)	
Deoli	2 (8.0%)	0 (0.0%)	
Akola	0 (0.0%)	1 (4.0%)	
Bramhapuri	1 (4.0%)	0 (0.0%)	
Darwha	0 (0.0%)	1 (4.0%)	
Kalamb	1 (4.0%)	0 (0.0%)	
Manora	0 (0.0%)	1 (4.0%)	
Nagpur	1 (4.0%)	0 (0.0%)	
Ralegaon	1 (4.0%)	0 (0.0%)	
Seloo	0 (0.0%)	1 (4.0%)	
Tiosa	1 (4.0%)	0 (0.0%)	
**District**			0.745 (2)
Wardha	20 (80.0%)	22 (88.0%)	
Yavatmal	2 (8.0%)	1 (4.0%)	
Akola	0 (0.0%)	1 (4.0%)	
Amravati	1 (4.0%)	0 (0.0%)	
Chandrapur	1 (4.0%)	0 (0.0%)	
Nagpur	1 (4.0%)	0 (0.0%)	
Washim	0 (0.0%)	1 (4.0%)	
**BMI (Kg/m^2^)**	17.74 ± 2.43	18.49 ± 2.08	0.243 (1)
**BMI**			0.769 (2)
< 18.5 Kg/m^2^	17 (68.0%)	15 (60.0%)	
18.5–22.9 Kg/m^2^	8 (32.0%)	9 (36.0%)	
23.0–24.9 Kg/m^2^	0 (0.0%)	1 (4.0%)	
**Occupation**			0.067 (2)
Farmer	12 (48.0%)	16 (64.0%)	
Housewife	2 (8.0%)	5 (20.0%)	
Student	5 (20.0%)	0 (0.0%)	
Mason	3 (12.0%)	1 (4.0%)	
Carpenter	1 (4.0%)	1 (4.0%)	
Barber	0 (0.0%)	1 (4.0%)	
Fisherman	0 (0.0%)	1 (4.0%)	
Fruit vendor	1 (4.0%)	0 (0.0%)	
Health care worker	1 (4.0%)	0 (0.0%)	
Smoking (yes)	8 (32.0%)	15 (60.0%)	0.047 (3)
Alcohol (yes)	6 (24.0%)	10 (40.0%)	0.225 (3)
Comorbidities: hypertension (yes)	0 (0.0%)	3 (12.0%)	0.235 (2)
Comorbidities: diabetes mellitus (yes)	2 (8.0%)	4 (16.0%)	0.667 (2)
Comorbidities: chronic kidney disease (yes)	0 (0.0%)	1 (4.0%)	1.000 (2)
Comorbidities: hypothyroidism (yes)	2 (8.0%)	0 (0.0%)	0.490 (2)
Comorbidities: chronic liver disease (yes)	0 (0.0%)	1 (4.0%)	1.000 (2)
Comorbidities: HIV (yes)	1 (4.0%)	2 (8.0%)	1.000 (2)
**Other risk factors: past history of tuberculosis**			0.714 (2)
Absent	17 (68.0%)	14 (56.0%)	
Present- treatment completed	5 (20.0%)	7 (28.0%)	
Present-defaulter	3 (12.0%)	4 (16.0%)	
**Other risk factors: contact history of tuberculosis (present)**	3 (12.0%)	0 (0.0%)	0.235 (2)
**Treatment duration**			< 0.001 (3)
9 months	25 (100.0%)	0 (0.0%)	
18 months	0 (0.0%)	25 (100.0%)	
Smear microscopy (3^rd^ month) (positive)	9 (36.0%)	5 (20.0%)	0.208 (3)
Smear microscopy (4^th^ month) (positive)	8 (32.0%)	4 (16.0%)	0.185 (3)
Smear microscopy (5^th^ month) (positive)	6 (24.0%)	2 (8.0%)	0.247 (2)
Smear microscopy (6^th^ month) (positive)	5 (20.0%)	2 (8.0%)	0.417 (2)
Culture (3^rd^ month) (positive)	7 (28.0%)	6 (24.0%)	0.747 (3)
Culture (6^th^ month) (positive)	5 (20.0%)	4 (16.0%)	1.000 (2)
Culture (9^th^ month) (positive)	3 (12.0%)	2 (8.0%)	1.000 (2)
Culture (12^th^ month) (positive)	0 (NaN%)	2 (8.0%)	1.000 (3)
Culture (18^th^ month) (positive)	0 (NaN%)	3 (12.0%)	1.000 (3)
ADRs: anaphylaxis (present)	0 (0.0%)	0 (0.0%)	1.000 (3)
ADRs: hepatitis (present)	4 (16.0%)	1 (4.0%)	0.349 (2)
ADRs: cardiac (present)	0 (0.0%)	1 (4.0%)	1.000 (2)
ADRs: neurological (present)	0 (0.0%)	2 (8.0%)	0.490 (2)
ADRs: optic neuritis (present)	0 (0.0%)	0 (0.0%)	1.000 (3)
ADRs: psychiatric (present)	2 (8.0%)	2 (8.0%)	1.000 (2)
ADRs: gastrointestinal (present)	7 (28.0%)	6 (24.0%)	0.747 (3)
ADRs: peripheral neuropathy (present)	1 (4.0%)	3 (12.0%)	0.609 (2)
ADRs: hematological (present)	0 (0.0%)	2 (8.0%)	0.490 (2)
ADRs: hypothyroidism (present)	1 (4.0%)	0 (0.0%)	1.000 (2)
ADRs: arthralgia (present)	5 (20.0%)	0 (0.0%)	0.050 (2)
**Interim outcome**			1.000 (2)
Bacteriological conversion	22 (88.0%)	22 (88.0%)	
Bacteriological reversion	2 (8.0%)	1 (4.0%)	
No Bacteriological conversion	1 (4.0%)	2 (8.0%)	
**Final outcome**			1.000 (2)
Cured	22 (88.0%)	21 (84.0%)	
Treatment failure	3 (12.0%)	3 (12.0%)	
Lost to follow up	0 (0.0%)	1 (4.0%)	

## 3 Results

1. The study included 50 Multidrug-resistant pulmonary Tuberculosis patients, who were evenly split between shorter and longer regimens based on the inclusion criteria.
2. The mean age was 41.44 years, and most (56%) were aged 20–40 years.
3. In the study, 70% of the participants were male and 30% were female.
4. The study showed that 64% of the participants were underweight (BMI < 18.5 kg/m^2^).
5. In the study, 12% of the patients had diabetes mellitus, 6% had HIV, and 6% had hypertension.
6. Alcohol consumption was associated with poor smear and culture conversion at the 3^rd^ month, with a significant impact on the final outcome.
7. Moderate association was seen between diabetes mellitus and delayed smear and culture conversion at the 3^rd^ month.
8. HIV infection was highly predictive of poor outcomes; all had no bacteriological conversion and experienced treatment failure.
9. It was found that 36% of the participants in the shorter regimen and 20% of the participants in the longer regimen were smear positive at the 3^rd^ month. A total of 20% in the shorter regimen and 8% in the longer regimen were smear positive at the 6^th^ month.
10. During the study, 28% of the participants in the shorter regimen and 24% of the participants in the longer regimen were culture positive at the 3^rd^ month. A total of 12% in the shorter regimen and 8% in the longer regimen were culture positive at the 9^th^ month.
11. All (100%) of the participants in the shorter regimen and 96% of the participants in the longer regimen were adherent to treatment. A total of 4% of the participants in the longer regimen were lost to follow-up.
12. In the study, 28% of the participants in the shorter regimen and 24% of the participants in the longer regimen had gastrointestinal side effects. A total of 20% of the participants in the shorter regimen had arthralgia. A total of 16% of the participants in the shorter regimen and 4% of the participants in the longer regimen had hepatitis. A total of 12% of the participants in the longer regimen had peripheral neuropathy.
13. At the end of the study, 88% of the participants in both the shorter and longer regimen had bacteriological conversion at the end of treatment. Bacteriological conversion was seen in 8% in the shorter regimen and 4% in the longer regimen. No bacteriological conversion was seen in 4% in the shorter regimen and 8% in the longer regimen.
14. The study concluded that 88% of the participants in the shorter regimen and 84% of the participants in the longer regimen were cured. Treatment failure was seen in 12% of the participants.

## 4 Discussion

Multidrug-resistant tuberculosis (MDR-TB) continues to be a major global health concern, threatening TB control efforts. It is caused by Mycobacterium tuberculosis, which is resistant to both isoniazid and rifampicin, requiring tailored treatment approaches. To address the complexities of MDR-TB management, programmatic management of drug-resistant tuberculosis (PMDT) strategies has been developed to improve treatment outcomes (7).

Programmatic management of drug-resistant tuberculosis (PMDT) is a coordinated and systematic strategy that combines diagnostic services, treatment protocols, and patient support within national TB control efforts. Its goal is to improve the quality and availability of care for people affected by drug-resistant TB through standardized and effective treatment approaches (8). Hence, the present study was conducted to compare the efficacy of longer versus shorter treatment regimens for multidrug-resistant tuberculosis (MDR-TB) patients under programmatic management of drug-resistant tuberculosis (PMDT).

The present study included all new MDR-TB patients and those already undergoing treatment at our institute. Participants were divided into two groups: one receiving a longer treatment regimen and the other receiving a shorter regimen. Follow-up involved monthly sputum smear examinations starting from the third month, as well as culture tests at the end of the third, sixth, and ninth months, and/or at the end of treatment. The two groups were compared in terms of treatment outcomes, drug compliance, and adverse effects.

In the present study, the mean age of study participants was 41.44 years (SD = 14.78), with a median of 37 years (IQR = 31–49.75) and a range of 20–77 years. The age distribution was: 56% between 20 and 40 years, 34% between 41 and 60 years, and 10% above 60 years. The gender distribution was 70% males and 30% females. Similar to the present study, Munir et al. (9) found that among 131 patients, 71 (54.2%) were males and 60 (45.8%) were females, the mean age of the patients was 35.08 ± 15.25. Another study by Ali et al. (10) reported that the gender distribution was 47.36% males and 52.63% females in the shorter treatment regimen (STR) group, and 55.42% males and 44.57% females in the longer treatment regimen (LTR) group. The mean age was significantly lower in the STR group (34.16 years, ± 16.76) compared to the LTR group (41.71 years, ± 18.44). A similar study by Kumari et al. (11) among the study subjects, 268 were males and 92 were females, median age was 41 years. Mansoori et al. (12) claimed the average age was 47 ± 19.06 years, and males were 788 (72.8%).

In the present study, as per distribution of the Participants in Terms of Taluk, 22.0% of the participants were from Wardha, 18.0% of the participants were from Samudrapur, 12.0% of the participants were from Arvi, 12.0% of the participants were from Hinganghat, 8.0% of the participants were from Karanja, 6.0% of the participants were from Ashti, 4.0% of the participants were from Deoli, 2.0% of the participants were from Akola, 2.0% of the participants were from Bramhapuri, 2.0% of the participants were from Darwha, 2.0% of the participants were from Kalamb, 2.0% of the participants were from Manora, 2.0% of the participants were from Nagpur, 2.0% of the participants were from Ralegaon, 2.0% of the participants were from Seloo and 2.0% of the participants were from Tiosa. A total of 84.0% of the participants were from district Wardha, 6.0% of the participants were from Yavatmal, 2.0% of the participants were from Akola, 2.0% of the participants were from Amravati, 2.0% of the participants were from Chandrapur, 2.0% of the participants were from district Nagpur and 2.0% of the participants were from Washim.

### 4.1 Distribution of the participants in terms of body mass index

In the study, 64.0% of the participants had a BMI < 18.5 Kg/m^2^, 34.0% of the participants had a BMI: 18.5–22.9 Kg/m^2^, and 2.0% of the participants had a BMI: 23.0–24.9 Kg/m^2^. In a similar study by Kumari et al. (11) 2.8% (10/360) of patients had a BMI < 18. A study by Prajapati et al. (13) reported body mass index > 18.5 as one of the factors for successful treatment outcomes of MDR TB.

### 4.2 Distribution of the participants in terms of occupation

Amongst the participants, 56.0% were farmers, 14.0% were housewives, 10.0% were students, 8.0% were masons, 4.0% were carpenters, 2.0% were barbers, 2.0% were fishermen, 2.0% were fruit vendors, and 2.0% were health care workers. In the study by Mansoori et al. (12) the study participant’s occupations varied, with a significant proportion being unemployed or disabled (34.1%), followed by housewives (23%), and daily laborers (20.6%).

### 4.3 Distribution of the participants in terms of smoking and alcohol consumption

In the present study, 46.0% of the participants were smokers, while 54.0% of the participants were non-smokers. A total of 32.0% of the participants were alcoholics, while 68.0% of the participants were non-alcoholics. In the study by Ali et al. (10) 61 (34.27%) of study participants were smokers. Mansoori et al. (12) reported that 48.1% of patients had a history of smoking. In a similar study by Prajapati et al. (13) among 112 patients, 35 had a single habit, which included 25 smokers, five alcohol consumers, and those who chewed tobacco. Additionally, 40 patients had multiple habits, combining tobacco chewing, smoking, and alcohol consumption, and this study revealed that tobacco chewing and alcohol consumption were linked to poorer treatment outcomes.

### 4.4 Distribution of the participants in terms of comorbidities

The participants in the study had various comorbidities, with Diabetes Mellitus being the most common, affecting 12% of the participants (six individuals). Hypertension and HIV were also notable, each affecting 6% of the participants (three individuals each). Additionally, 4% of the participants (two individuals) had Hypothyroidism, while chronic kidney disease and Chronic Liver Disease were each present in 2% of the participants (one individual each). In a similar study by Kumari et al. (11) 10.28% (37/360) of study participants were diabetics, and 13.5% (47/360) cases were HIV reactive. In the study by Mansoori et al. (12) comorbidities included diabetes in 14.1% of patients. A study by Mphande-Nyasulu et al. (14) found that individuals with comorbidities had a 2.16-fold increased risk of TB compared to those without comorbidities, although this finding was not statistically significant (95% CI: 0.33–13.98). Patients with HIV infection had a 2.22-fold increased risk of TB (95% CI: 0.93–5.31). While these findings suggest potential associations, further research is needed to confirm these relationships.

### 4.5 Distribution of the participants in terms of past history of tuberculosis

In the study, 62.0% of the participants had no history of tuberculosis. A total of 24.0% of the participants had a history of tuberculosis who completed the treatment, while 14.0% of the participants had a history of tuberculosis but were defaulters. Mansoori et al. (12) reported that among patients with MDR tuberculosis, a total of 156 patients (19.5%) had a history of treatment with anti-tubercular therapy.

### 4.6 Distribution of the participants in terms of contact history of tuberculosis

Amongst the participants, 6.0% had a contact history of tuberculosis, while 94.0% had no contact history of tuberculosis.

### 4.7 Distribution of the participants in terms of “smear microscopy and culture results”

The study’s findings showed that at the 3^rd^ month, 28% of participants tested positive on smear microscopy, while 72% tested negative. By the 6^th^ month, the positivity rate had decreased to 14%, with 86% testing negative. Culture results also showed a decline in positivity rates over time, with 26% testing positive at 3 months and 74% negative. At 9 months, 10% were culture-positive and 90% were negative, and at 18 months, 12% were positive and 88% were negative. These results indicate a general trend of decreasing positivity rates for both smear microscopy and culture tests over the course of treatment. The above findings suggest that treatment is effective in reducing the bacterial load over time, as evidenced by the decline in positivity rates for both smear microscopy and culture tests. The decrease in positivity rates from 3 to 6 months for smear microscopy and from 3 to 9 months for culture tests indicates a positive response to treatment. However, the slight increase in culture positivity at 18 months compared to 9 months suggests bacteriological reversion. Overall, the results suggest that the treatment regimen is effective in achieving a bacteriological response in a significant proportion of patients. In the study by Prajapati et al. (13) out of 112 patients, 61 (54%) achieved sputum culture negativity by 12 months, with 49 of them converting within the first 6 months. Successful treatment outcomes were observed in 29 patients (25.89%), and this study reported that factors associated with favorable treatment outcomes included age ≤ 40 years, body mass index > 18.5, and sputum/culture conversion at 3 months, and tobacco chewing and alcohol consumption were linked to poorer treatment outcomes.

### 4.8 Distribution of the participants in terms of adverse drug reactions

In the present study, gastrointestinal issues were the most common, affecting 26% of participants, followed by arthralgia and hepatitis (10% each). The study by Ali et al. (10) found a much higher incidence of gastric irritation (56.18%), hepatitis (25.84%), and ototoxicity (19.10%). Nephrotoxicity was also more common in the Ali et al. (10) study (12.36%). Both studies reported psychiatric issues, but the frequency was much lower in the present study (8% vs. 3.93% for psychosis). Overall, the Ali et al. (10) study reported higher rates of adverse effects (Table 2).

**TABLE 2:** Distribution of the participants in terms of adverse drug reactions.

Adverse drug reactions	Frequency	Percentage
Anaphylaxis	0	0.0%
Hepatitis	5	10.0%
Cardiac	1	2.0%
Neurological	2	4.0%
Optic neuritis	0	0.0%
Psychiatric	4	8.0%
Gastrointestinal	13	26.0%
Peripheral neuropathy	4	8.0%
Hematological	2	4.0%
Hypothyroidism	1	2.0%
Arthralgia	5	10.0%

### 4.9 Distribution of the participants in terms of “interim outcome and final outcome”

In the study, 88.0% of the participants had bacteriological conversion, 6.0% of the participants had bacteriological reversion, and 6.0% of the participants had no bacteriological conversion. It was found that 86.0% of the participants were cured, 12.0% experienced treatment failure, and 2.0% were lost to follow-up. Various studies have reported treatment success rates for MDR-TB patients. Ali et al. (10) found that the shorter regimen (STR) group had a higher treatment success rate of 86.31% compared to the long-term regimen (LTR) group at 79.51%. Similarly, Abidi et al. (15) reported a higher success rate for shorter regimens at 80.0% versus 75.3% for longer regimens. Other studies have also shown promising results, with Wakjira et al. (16) reporting a 69% successful treatment completion rate, and Panford et al. (17) finding 71.7% successful treatment outcomes in a Ghanaian study. Wahid et al. (18) observed a treatment success rate of 83.7% in the STR group and 73.2% in the LTR group. Trubnikov et al. (19) reported 66.3% successful treatment outcomes, while Kumari et al. (11) found a cure rate of 42.50% and treatment completion rate of 41.60%, resulting in an overall treatment success rate of 84.1%. Munir et al. (9) found that the overall cure rate was 74.8% (98 patients), with 13.8% of patients completing treatment successfully. These studies highlight the varying degrees of success in treating MDR-TB with different regimens.

### 4.10 Association of alcohol consumption with smear microscopy and culture

The present study found that alcohol consumption is linked to poorer tuberculosis treatment outcomes, with higher rates of positive smear microscopy and culture results. Alcohol consumption also affects final treatment outcomes, with lower cure rates (68.8% vs. 94.1%).

The present study found a significant association between alcohol consumption and smear microscopy results at the 3^rd^ month (χ2 = 9.314, *p* = 0.005). A larger proportion of participants in the alcoholic group had positive smear microscopy results (56.2%) compared to the non-alcoholic group (14.7%). A larger proportion of participants in the alcoholic group had positive culture results (62.5%) compared to the non-alcoholic group (8.8%). At the 9^th^ month, there is a significant association between alcohol consumption and culture results (χ2 = 5.882, *p* = 0.031) with a moderate strength of association (Cramer’s V = 0.34). A larger proportion of participants in the alcoholic group had positive culture results (25.0%) compared to the non-alcoholic group (2.9%). At the 18^th^ month, there is no significant association between alcohol consumption and culture results (χ2 = 0.063, *p* = 1.000). Similarly, in the study by Duraisamy et al. (20), among the patients who failed to achieve sputum culture conversion, 33% consumed alcohol during treatment. In one case, the patient had only missed two doses, suggesting that alcohol’s effects may have contributed to treatment failure rather than non-adherence or ineffective treatment. Another study by Deshmukh et al. (21) revealed that alcohol consumption resulted in missed anti-tubercular treatment doses, missed medical appointments, and patients were not willing to undergo counseling. Various studies by Oeltmann et al. (22), Rehm et al. (23), Jakubowiak et al. (24), Parry et al. (25) has consistently shown that alcohol abuse is linked to poor treatment compliance and clinical outcomes in various diseases, including tuberculosis and these studies have found that individuals who abuse alcohol are more likely to default from TB treatment. Jakubowiak et al. (24), Driver et al. (26) reported that excessive alcohol consumption is associated with a higher likelihood of treatment interruptions, which can negatively impact treatment outcomes. Missed doses likely contribute to most unsuccessful treatment outcomes. However, some patients who consumed alcohol while adhering to treatment still experienced poor outcomes. Alcohol abuse can impact the immunity of the patient, directly leading to treatment failure or poor response (20). The present study found a significant association between alcohol consumption and final outcome (χ2 = 6.253, *p* = 0.027). A larger proportion of participants in the non-alcoholic group were cured (94.1%) compared to the alcoholic group (68.8%). Conversely, a larger proportion of participants in the alcoholic group had treatment failure (25.0%) and were lost to follow-up (6.2%). The results are similar to the study conducted by Duraisamy et al. (20) which found that patients who consumed alcohol during treatment were 4.3 times more likely to have unsuccessful outcomes. They also missed an average of 18 more doses during the intensive phase compared to non-drinkers.

Normally, alveolar macrophages eliminate over 90% of inhaled M. tuberculosis bacteria in immunocompetent individuals (27). Studies have shown that alcohol can enhance the survival of mycobacteria within the macrophages by suppressing key immune functions, including mobilization, adherence, phagocytosis, and superoxide production (28, 29). Alcohol consumption has been found to impair antigen-specific T-cell activation by disrupting the presentation of mycobacterial antigens to lymphocytes. Chronic alcohol exposure can also suppress cytokine production, which plays a crucial role in cellular communication, activation, and regulation of inflammation and healing mechanisms (29).

### 4.11 Association of “comorbidities: diabetes mellitus” with smear microscopy and culture

There is a significant association between diabetes mellitus and smear microscopy results at the 3^rd^ month (χ2 = 5.057, p = 0.044) with a moderate strength of association (Cramer’s V = 0.32). A larger proportion of diabetic participants (66.7%) had positive smear microscopy results compared to non-diabetics (22.7%). At the 6^th^ month, there is no significant association between diabetes mellitus and smear microscopy results (χ2 = 2.117, *p* = 0.192) with a low strength of association (Cramer’s V = 0.21).

In the present study, a significant association between diabetes mellitus and culture results at the 3^rd^ month (χ2 = 5.861, *p* = 0.033) with a moderate strength of association (Cramer’s V = 0.34). A larger proportion of diabetic participants (66.7%) had positive culture results compared to non-diabetics (20.5%). At the 9^th^ month, there is no significant association between diabetes mellitus and culture results (χ2 = 0.758, *p* = 1.000), with little to no association (Cramer’s V = 0.12). None of the diabetic participants had positive culture results. At the 18^th^ month, there is no significant association between diabetes mellitus and culture results (χ2 = 0.649, *p* = 1.000), with little to no association (Cramer’s V = 0.16). None of the diabetic participants had positive culture results. The study by Viswanathan et al. (30) showed that patients with diabetes (TB-DM) had delayed sputum conversion and higher treatment failure rates compared to non-diabetic patients (TB non-DM). Specifically, 14.7% of TBDM patients remained sputum-positive at the end of the intensive phase, compared to 3.5% of TB non-DM patients. The mean duration for sputum conversion was also longer in TBDM patients (64.2 days) compared to TB non-DM patients (61.5 days).

The present study found no significant association between diabetes mellitus and interim outcome (χ2 = 0.930, *p* = 1.000) with a low strength of association (Cramer’s V = 0.14). All diabetic participants had bacteriological conversion. There is no significant association between diabetes mellitus and final outcome (χ2 = 0.266, *p* = 0.616), with little to no association (Cramer’s V = 0.07). The proportions of participants who were cured (83.3% vs. 86.4%) and experienced treatment failure (16.7% vs. 11.4%) were similar between the diabetic and non-diabetic groups. A study by Duraisamy et al. (20) reported that the prevalence of diabetes (33%) did not significantly impact treatment outcomes. Another study by Nandakumar et al. (31) found that while patients with unknown DM status and those with DM had higher rates of unfavorable outcomes (23% and 17%, respectively), the difference was not statistically significant, however, poor glycemic control or unknown control status showed a trend toward increased risk of unfavorable outcomes. Xu et al. (32) found that diabetes mellitus (DM) was significantly associated with adverse treatment outcomes. Specifically, DR-TB patients with DM had a higher risk of unsuccessful treatment outcomes, treatment failure, and lower rates of cure and treatment completion. Similar associations were observed in MDR-TB patients. In a study by Viswanathan et al. (30) TBDM patients had higher rates of treatment failure (4.2% vs. 0.7%) and multidrug-resistant TB (1%), and similarly, in the present study, it was concluded that diabetes is associated with poorer TB treatment outcomes and increased treatment failure.

### 4.12 Association of “comorbidities: HIV” with smear microscopy and culture

The present study reveals a significant and strong association between HIV and poorer tuberculosis treatment outcomes. Participants with HIV had higher rates of positive smear microscopy and culture results, and lower rates of bacteriological conversion and cure. Notably, all participants with HIV had treatment failure, whereas the majority of non-HIV participants were cured. These findings suggest that HIV co-infection significantly impacts tuberculosis treatment outcomes, highlighting the need for targeted interventions and management strategies for individuals with HIV-TB co-infection.

In the present study, a significant association between HIV and smear microscopy results at the 3^rd^ month (χ2 = 8.207, *p* = 0.019) with a moderate strength of association (Cramer’s V = 0.41). A total of 100% of participants with HIV had positive smear microscopy results at the 3rd month compared to 23.4% of non-HIV participants. At the 6^th^ month, there is a significant association between HIV and smear microscopy results (χ2 = 19.605, *p* = 0.002) with a high strength of association (Cramer’s V = 0.63). A total of 100% of participants with HIV had positive smear microscopy results at the 6^th^ month compared to 8.5% of non-HIV participants. A study by Singh et al. (33) indicated that individuals living with HIV who are co-infected with multidrug-resistant/extensively drug-resistant tuberculosis (M/XDR-TB) experience poor outcomes and high mortality rates. This co-infection is also linked to M/XDR-TB epidemics and outbreaks.

The present study found a significant association between HIV and culture results at the 3^rd^ month (χ2 = 9.083, *p* = 0.015) with a moderate strength of association (Cramer’s V = 0.43). A total of 100% of participants with HIV had positive culture results at the 3^rd^ month compared to 21.3% of non-HIV participants. At the 9^th^ month, there is a significant association between HIV and culture results (χ2 = 11.387, *p* = 0.023) with a moderate strength of association (Cramer’s V = 0.48). A total of 66.7% of participants with HIV had positive culture results at the 9^th^ month compared to 6.4% of non-HIV participants. At the 18^th^ month, there is a significant association between HIV and culture results (χ2 = 15.942, *p* = 0.010) with a high strength of association (Cramer’s V = 0.8). A total of 100% of participants with HIV had positive culture results at the 18^th^ month compared to 4.3% of non-HIV participants.

In the present study, there was a significant association between HIV and interim outcome (χ2 = 26.359, *p* = 0.001) with a high strength of association (Cramer’s V = 0.73). None of the participants with HIV had bacteriological conversion, while 93.6% of non-HIV participants had bacteriological conversion. The study by Isaakidis et al. (34) revealed that among HIV patients who had previously received treatment for TB, had a higher proportion of multidrug-resistant TB and more advanced TB resistance profiles (36%) compared to those who were newly diagnosed (11%).

In the present study, there was a significant association between HIV and final outcome (χ2 = 23.404, *p* = 0.002) with a high strength of association (Cramer’s V = 0.68). A total of 100% of participants with HIV had treatment failure, while 91.5% of non-HIV participants were cured. The study by Chikkahonnaiah (35) reported that the clinical stage of HIV significantly influenced treatment outcomes. Patients with stage II HIV had a higher cure rate of 55.6% (10 patients) and a lower mortality rate of 16% (4 patients). In contrast, patients with stage IV HIV had a mortality rate of 72% (18 patients). This association was statistically significant, with a *p*-value of 0.002.

### 4.13 Comparison between longer and shorter regimens with smear microscopy and culture

In the present study, there was no significant association between treatment regimen (Shorter vs Longer) and smear microscopy results at the 3^rd^ month (χ2 = 1.587, *p* = 0.208) or 6^th^ month (χ2 = 1.495, *p* = 0.417). Similarly, there is no significant association between treatment regimen and culture results at the 3^rd^ month (χ2 = 0.104, *p* = 0.747) or 9^th^ month (χ2 = 0.222, *p* = 1.000). The strength of association between treatment regimen and smear microscopy and culture results is low (Cramer’s V < 0.2). The proportions of participants with positive and negative smear microscopy and culture results are comparable between the two treatment regimens. In a similar study by Wahid et al. (18) patients received either a shorter treatment regimen (STR) or a longer treatment regimen (LTR). The STR group had a significantly shorter time to sputum culture conversion (SCC) compared to the LTR group (2.03 vs. 2.69 months, *p* < 0.001). Multivariate analysis showed that STR was the only predictor of early SCC, while factors like older age, lower baseline body weight, and LTR treatment were associated with unsuccessful treatment outcomes and their study concluded that STR demonstrated superior antimicrobial activity against MDR-TB, resulting in earlier SCC, higher cure rates, and lower mortality compared to LTR.

### 4.14 Comparison of adverse drug reactions in the shorter regimen and the longer regimen

The safety and tolerability of shorter MDR-TB regimens have been debated. A study by Trubnikov et al. (19) in Uzbekistan, found healthcare providers were skeptical about the regimen’s safety due to potential toxicity concerns. However, a systematic review and meta-analysis by Nyang’wa et al. (36) suggested shorter regimens had a lower risk of loss to follow-up compared to longer ones. The present study compared the adverse drug reactions (ADRs) between patients receiving a shorter regimen and those receiving a longer regimen (Table 3). No significant differences were observed in the incidence of most ADRs between the two groups. Gastrointestinal ADRs were common, affecting 28% of patients in the shorter regimen group and 24% of patients in the longer regimen group. Arthralgia was significantly more common in the shorter regimen group (20%) compared to the longer regimen group (0%). Hepatitis, neurological, and hematological ADRs were also observed, but the differences between the groups were not statistically significant. In contrast, the study by Ali et al. (10) found significant differences in the incidence of certain ADRs, with gastric irritation (63.16%) and skin pigmentation (11.58%) in the shorter regimen group, whereas those in the longer regimen group were more likely to experience myelosuppression (13.68%). These disparate findings highlight the need for further research to understand the relationship between treatment duration and ADR. These findings imply that treatment duration may influence the type and frequency of adverse effects, which could inform treatment decisions and monitoring strategies.

**TABLE 3:** A comparison of adverse drug reactions in the shorter regimen and the longer regimen.

Adverse drug reactions	Treatment		*P*-value
	Shorter regimen (*n* = 25)	Longer regimen (*n* = 25)	
Anaphylaxis	0 (0.0%)	0 (0.0%)	1.000 (3)
Hepatitis	4 (16.0%)	1 (4.0%)	0.349 (2)
Cardiac	0 (0.0%)	1 (4.0%)	1.000 (2)
Neurological	0 (0.0%)	2 (8.0%)	0.490 (2)
Optic neuritis	0 (0.0%)	0 (0.0%)	1.000 (3)
Psychiatric	2 (8.0%)	2 (8.0%)	1.000 (2)
Gastrointestinal	7 (28.0%)	6 (24.0%)	0.747 (3)
Peripheral neuropathy	1 (4.0%)	3 (12.0%)	0.609 (2)
Hematological	0 (0.0%)	2 (8.0%)	0.490 (2)
Hypothyroidism	1 (4.0%)	0 (0.0%)	1.000 (2)
Arthralgia	5 (20.0%)	0 (0.0%)	0.050 (2)

In the present study shorter regimen group, the most common ADRs were gastrointestinal (28%), arthralgia (20%), and hepatitis (16%). Some patients experienced psychiatric ADRs (8%), peripheral neuropathy (4%), and hypothyroidism (4%). No patients experienced anaphylaxis, cardiac, or optic neuritis. In the longer regimen group, the most common ADRs were gastrointestinal (24%), neurological (8%), and psychiatric (8%). Some patients experienced peripheral neuropathy (12%), hematological ADRs (8%), and cardiac ADRs (4%). No patients experienced anaphylaxis or optic neuritis. In a similar study by Munir et al. (9) it was found that the shorter regimen group experienced significantly fewer side effects at treatment completion compared to the longer regimen group (*p* < 0.05). In contrast, a study by Trubnikov et al. (19) during the treatment period, 47 drug adverse events (DAEs) were reported, resulting in an incidence rate of 6.15 DAEs per 100 person-months. A total of 38 patients (40%) experienced at least one DAE, with 21 patients (22.1%) experiencing grade 3 or 4 DAEs. The median time to onset of DAEs was 101 days (64–139 days). The common DAEs were gastrointestinal disorders, liver failure, and ototoxicity, with Prothionamide being the most frequently implicated drug. In response to DAEs, treatment adjustments were made, including temporary interruption of the offending drug in 55.3% of cases, dose reduction in 8.5%, and permanent discontinuation in 8.5% of cases. HIV emerged as the only predictor correlated with a greater hazard of DAE.

### 4.15 Treatment adherence

The adherence to treatment in the shorter regimen was 100% and 96% in the longer regimen. The high adherence rate observed in both the shorter and longer regimen groups suggests that patients were generally able to comply with their prescribed treatment schedules, likely supported by structured follow-up, patient counseling, and access to directly observed therapy or similar adherence support strategies. The slightly lower adherence in the longer regimen could reflect challenges in maintaining prolonged treatment, including side effects, fatigue, or loss of motivation. Despite high adherence rates overall, monitoring remains crucial, especially for longer regimens, to prevent treatment failure or the development of further resistance. These findings support the potential public health benefit of implementing shorter regimens in national TB control programs to enhance adherence and outcomes.

### 4.16 Association between “treatment” and “interim outcome”

Both treatment regimens have similar efficacy in achieving bacteriological conversion, and the treatment regimen may not significantly impact interim outcomes (Table 4). The study found no significant association between treatment regimen and interim outcome (χ2 = 0.667, *p* = 1.000), with 88% of participants in both the shorter and longer regimen groups achieving bacteriological conversion. The proportions of participants with bacteriological reversion (8% vs. 4%) and no bacteriological conversion (4% vs. 8%) were also similar between the two groups. The strength of association between treatment regimen and interim outcome was low (Cramer’s V = 0.12), suggesting that the treatment regimen may not have a significant impact on interim outcomes.

**TABLE 4:** The association between “treatment” and “interim outcome.”

Interim outcome	Treatment			Fisher’s exact test	
	Shorter regimen	Longer regimen	Total	χ 2	*P*-value
Bacteriological conversion	22 (88.0%)	22 (88.0%)	44 (88.0%)	0.667	1.000
Bacteriological reversion	2 (8.0%)	1 (4.0%)	3 (6.0%)		
No bacteriological conversion	1 (4.0%)	2 (8.0%)	3 (6.0%)		
Total	25 (100.0%)	25 (100.0%)	50 (100.0%)		

### 4.17 Association between “treatment” and “final outcome”

The present study investigated the association between treatment regimen and final outcome, utilizing Fisher’s exact test, and the results indicated no significant difference in the distribution of final outcomes between the two treatment groups, with a chi-squared value of 1.023 and a *p*-value of 1.000. In terms of specific outcomes, 88.0% of participants who received the shorter regimen were cured, while 12.0% experienced treatment failure, and none were lost to follow-up. Similarly, 84.0% of participants who received the longer regimen were cured, 12.0% had treatment failure, and 4.0% were lost to follow-up (Table 5). The study suggests that both treatment regimens have comparable efficacy in achieving a cure, with a high proportion of participants being cured in both groups (88% in the shorter regimen group and 84% in the longer regimen group). The similarity in treatment failure rates (12% in both groups) further supports the notion that the treatment regimens have similar outcomes. Overall, the findings indicate that the shorter regimen may be as effective as the longer regimen in achieving favorable treatment outcomes. Our results are consistent with Munir et al. (9) that reported cure rates were comparable between the longer regimen (75.6%) and shorter regimen (73.8%) treatment groups, with similar treatment failure rates (6.2% and 6.1%, respectively), and these differences in treatment outcomes were not statistically significant (*p* > 0.05). Another study by Ali et al. (10) compared treatment outcomes between shorter regimen (STR) and longer regimen (LTR) groups. The STR group had a higher treatment success rate of 86.31% (82 patients) and a lower death rate of 4.21% (4 patients), whereas the LTR group had a success rate of 79.51% (66 patients) and a higher death rate of 9.63% (8 patients). Another study by Abidi et al. (15) also revealed that the shorter regimen had higher success rates with pooled proportions of 80.0% versus 75.3% and reported that the difference was attributed to fewer patients being lost to follow-up in the shorter regimen group and may even improve adherence and patient outcomes primarily due to reduced loss to follow-up in the shorter regimen group. A study by Wakjira et al. (16) found that after 24 months, 69% of patients had completed treatment, while 27% had died from the disease. A retrospective cohort study by Lecai et al. (37) in China highlighted the challenge of adverse events, with 24.9% of patients requiring a modification in MDR-TB treatment due to these events, though the success rate was not specified. In Ghana, a study conducted by Panford et al. (17) showed that 71.7% of patients achieved successful treatment outcomes for MDR-TB, although a mortality rate of 17.0% was observed. In a similar study by Wahid et al. (18) treatment success was higher in the STR group (83.7% vs. 73.2%, *p* < 0.001), attributed to higher cure rates (79.9% vs. 70.9%, *p* = 0.006) and lower death rates (9.9% vs. 18.3%, *p* = 0.002). A study by Trubnikov et al. (18) evaluated the effectiveness and predictors of drug adverse events (DAEs) in patients treated with a shorter treatment regimen (STR) for rifampicin-resistant tuberculosis and treatment outcomes showed that 66.3% of patients were successfully treated, while 17.9% experienced treatment failure, 7.4% died, 5.3% were lost to follow-up, and 3.2% were not assessed. Among the 54 patients who completed treatment, no recurrence was detected after 12 months. In another study by Kumari et al. (11) out of 360 confirmed MDR-TB patients, 42.5%, were cured, and 41.60% completed treatment, resulting in an overall treatment success rate. However, 6.11% were lost to follow-up, 0.50% experienced treatment failure, and 9.10% died. The study concluded that the standardized shorter MDR regimen showed high overall success rates with low treatment failure rates; however, the cure rate was lower than this study.

**TABLE 5:** The association between “treatment” and “final outcome.”

Final outcome	Treatment			Fisher’s exact test	
	Shorter regimen	Longer regimen	Total	χ 2	*P*-value
Cured	22 (88.0%)	21 (84.0%)	43 (86.0%)	1.023	1.000
Treatment failure	3 (12.0%)	3 (12.0%)	6 (12.0%)		
Lost to follow up	0 (0.0%)	1 (4.0%)	1 (2.0%)		
Total	25 (100.0%)	25 (100.0%)	50 (100.0%)		

These findings suggest that shorter regimens can be as effective as longer regimens, often due to lower rates of loss to follow-up (38). However, the potential for adverse events necessitates vigilant management to ensure optimal treatment outcomes. The above literature suggests that shorter treatment regimens may be as effective as longer ones for some drug-resistant TB patients, potentially reducing treatment burden and improving patient outcomes. The higher success rate in the STR group may be attributed to better adherence, a crucial factor in TB management.

## 5 Conclusion

The study found that both shorter and longer regimens for MDR-TB have comparable efficacy in achieving bacteriological conversion and cure. The treatment outcomes, including smear microscopy and culture results, were similar between the two groups. Notably, the shorter regimen group had a slightly higher cure rate (88%) compared to the longer regimen (84%). HIV co-infection, diabetes mellitus, and alcohol consumption were associated with poor treatment outcomes. Overall, the findings suggest that the shorter regimen may be as effective as the longer regimen in achieving favorable treatment outcomes, which could have significant implications for reducing treatment duration and improving patient compliance.

## 6 Limitations

The sample size was relatively small, which may limit the generalizability of the results to larger populations. Although both regimens had similar efficacy, the study may have been underpowered to detect clinically significant differences. Additionally, the study was conducted in a single center in a specific region, which may not represent other areas with different demographics, clinical characteristics, and healthcare infrastructure.

## 7 Recommendations

National TB programs should consider the use of shorter regimens for MDR-TB patients as they appear to be as effective as the longer regimens. With the introduction of newer regimens like BPaLM and BpaL, multicenter trials comparing them with the shorter regimen must be carried out. Targeted interventions should be implemented to address the needs of patients with HIV and diabetes mellitus, who are at higher risk of poor treatment outcomes. After prescribed treatment completion, follow-up culture tests should be done for a minimum of 2 years or when symptomatic to rule out any chance of recurrence. Therapeutic drug monitoring, host-directed immunotherapies, autophagy inducers, gene therapy, and RNA-based therapy are new areas of interest.

## Data Availability

The original contributions presented in this study are included in this article/supplementary material, further inquiries can be directed to the corresponding author.
